# Orientation to the patient as a marketing strategy in the Romanian healthcare system

**Published:** 2016

**Authors:** BI Coculescu, EC Coculescu, VL Purcărea

**Affiliations:** * “Titu Maiorescu” University, Faculty of Medicine; Center for Military Medical Scientific Research, Ministry of National Defence, Bucharest, Romania; ** “Carol Davila” University of Medicine and Pharmacy, Faculty of Dental Medicine, Bucharest, Romania; *** “Carol Davila” University of Medicine and Pharmacy, Faculty of Medicine, Bucharest, Romania

**Keywords:** Romanian public health system, medical marketing, patient-centered care

## Abstract

In the context of the European political and socio-economic changes of the early 90s, health care reform in Romania has become inevitable, both for patients and for health professionals in the system. The first stage of the health care reform in Romania is focused on decentralization and improving primary health care. The Romanian medical system is currently in the process of changing the mentality, which requires time, patience, and perseverance, despite the unforeseeable or resistance inevitably faced. It is a commonly known and recognized fact that in this painful period of transition, Romanian medicine, like other fields too, has traveled a winding road, with obstacles (medical malpractices, scandals in the press related to the misappropriation of funds or underfunding of the public health system, etc.) often hard to overcome.

## Introduction

In most countries, a national health policy establishes the way health care services can be accessed and finances these types of medical services provided to the population. The financing system represents the instrument for the implementation of this policy and the resources in order to include the collection of the resources for medical paid leaves, their allocation, and payment of production factors.

The settlement of medical services covered by social health insurance of the Health Insurance Houses is the main source of funding of the health service providers in Romania.

**Table 1:** SWOT analysis of the Romanian health system (Strengths, Weaknesses, Opportunities, Threats)

Strengths(S)	Weaknesses (W)
• the accessibility of health services	• underfunding of hospitals
• competent professionals in the field	• unequal distribution of resources
• most of the institutions in the system have operating permits.	• lack of therapeutic guidelines in most medical specialties
	• lack of a coherent health policy
	• lack of real competition in the system due to the low private developments.
Opportunities (O)	Threats(T)
• better access to EU funds	• economic crisis
• promoting research and development projects in the field	• exodus of health professionals
• promoting public-private partnership for the benefit of medical units (outsourcing medical analysis laboratories, cleaning services, laundry, kitchen)	• low level of medical training due to:
• use of renewable and alternative energies for hospitals	- poor academic selection of future doctors
• increasing the share of private medical establishments	- decreased interest in the craft itself in the context of mediocre pay scales for health professionals
• collaboration with the National Health Insurance House (county).	• blocking positions in the Government sector
	• lack of investment in the field.

Due to the medical services whose supply has a high value, a series of national programs, for which payment is made according to the main source of funding: the Ministry of Health, or the single fund health insurance, were provided in the healthcare system in Romania. Other sources of income within the public health system are the state budget revenues with special purpose (investment) and extra-budgetary revenues (e.g., renting, leasing premises, donations, and sponsorships).

The distribution of funds, collected through one of the mechanisms described above, is to the health sector (primary, secondary, tertiary), regionally, and to health care providers (hospitals, dispensaries, clinics, private offices).

## Discussion

The current stage of the concept of health system financing involves the collection of funds for the payment of health services, regional allocation of these funds to other service providers or health and medical staff remuneration. Among the sources that might supplement health spending, in line with the real needs of the population, are the increased number of insured persons in the mandatory health insurance system by eliminating exemptions from contribution payments, greater involvement of local communities in the financing of healthcare services covered by the payment health insurance and the increased funding from the state budget. Also, preventive services should also represent an important step of reform, to lower the costs in the healthcare sector, focusing on prevention as the key to success of any health care system. The system must focus on prevention and treatment of illnesses at their starting stage because this strategy requires less financial resources.

Expenses for medical assistance vary widely from country to country, 4-14% of the gross domestic product (GDP) of the country. States that spend less than 4% have a medical system of subsistence. As far as Romania is concerned, in the coming years it is necessary to raise the GDP to 6-7% so as the health system to become effective. In addition, unlike many Western countries, Romanians pay 25-30% of the money in the system out of their pockets (direct payment); while the Germans pay only 4%.

The medical system depends directly on financing, including the allocated percentage of the GDP and the rational manner in which this funding is used, this being the “engine” capable of conducting all the reforms which refer to the increase of efficiency and quality of healthcare, in terms of endowment with modern equipment and latest generation of drugs, while ensuring an adequate remuneration of the medical act.

Currently, the challenge for all those involved in the health care service system is how to respond to the five questions of client-oriented marketing, at the time of first contact with the client / patient / health service: Ask the client what he really wants; Focus on what the customer thinks is important; Make sure you can act on feedback from your client; Get feedback on an ongoing basis; Create feedback loops in your entire company [**1**].

Even in developed countries, authorized signals highlight the chronic deficiencies in the health care system: rising costs, lower customer satisfaction, growing deficiencies in quality, limited coverage [**2**].

For example, the current US health system (considered dysfunctional because although 16% of the GDP is allocated to health care, many patients have severe diseases) needs a change that can be measured by the health benefit per person/ dollar spent.

In order to increase the quality of the health services, a series of strategic organizational measures is required from the health care providers:

• analysis of the results, methods, experience in the medical practice units;

• read dress pricing methods by creating a single invoice;

• medical services based on excellence;

• uniqueness of medical investigation methods.

It was found that an increasing number of vendors have started to address several goals, but few are those who provide the full range of medical services, although this is beneficial for both parties.

Depending on the needs and expectations of the patient, medical services must provide both performance and quality. Providing a complex medical service has to meet the needs of patients. Through a well-run management of the quality of medical services, all the goals that satisfy and meet all the conditions of the beneficiaries of these services can be achieved.

It is known that no organization, no matter how efficient and innovative, can fully achieve the expectations of all the patients. It is therefore necessary to turn to those consumer segments that meet their needs as completely and complex as possible in order to ensure maximum satisfaction coupled with a considerable profit. Simultaneously, healthcare organizations must be constantly preoccupied with market developments to provide health care performance standards. Without taking into account what consumers really want, all that a medical organization can do is to cap quality services.

To attract the confidence of patients in the medical services provided, the specialist staff of the organizations in the field must be interested and involved in the issues expressed by the patients.

These goals can be met depending on how the organization responds to the following medical conditions:

• carry out an effective communication with the patients;

• present an objective picture of health services and provide the promised service properly;

• there is a constant concern to continuously improve the service provided to achieve patient expectations (i.e., the provision of excellent service).

The medical organizations must show a continuous concern in two directions: explicitly (i.e., a proper advertising, personal sales, etc.) and also implicitly (the price reflects the expectations of customers, presenting an accurate picture of the medical characteristics, etc.) to eliminate patient dissatisfaction. As such, healthcare organizations can become profitable and popular by mobilizing efforts to guarantee and fulfill the health service offers to patients. Achieving these goals should objectively reflect the service provided and not its “sanitized” version.

Obviously, marketing situations differ, but the organizations concerned about the essence of success grasp the strategic importance of aligning the relations between all those key interests in a medical institution - shareholders, managers, health professionals, patients - and the consequences of these interactions to improve the quality of the medical service and thereby the performance and profitability of the institution as such and even the relational landscape as a whole, transforming patients into real promoters of the brand, thus multiplying marketing efforts [**3**].

The feedback of satisfaction from the beneficiaries of health care services to improve the quality of services, involves carrying out concrete steps of action by the organization. These actions have their starting point at the first contact with the patient and run up to the end of the entire chain of medical services offered, which is reflected most often in handing the discharge documents. In this complex process of delivering health services, immediately after the patient’s first contact with the service provider, the patient assesses the quality of the service and the satisfaction felt thanks to it. If the patient’s assessment (subjective) is positive, or if the patient’s expectations have been met, he can develop a so-called loyalty to the healthcare organization. This behavior results in the patient’s confidence in the quality of the medical services provided by the respective organization. A loyal patient intents to reapply for health care services provided by the medical organization concerned [**4**]. A loyalty management to keep patients also has an effect on the volume of medical services performed and consequently on the turnover.

Medical organizations must base their work on the following considerations to provide superior healthcare benefits in terms of quality:

• developing very high performance standards of quality of its medical services;

• supervising the delivery of health services, so that they continuously analyze their own performances and those of their competitors through specific methods: fictional patient test, study of suggestions, complaints and opinions of patients, audit teams for medical services, etc.;

• constant involvement of the management in the issue of quality through a monthly analysis of not only financial results but also of the quality level of the medical services provided;

• creating conditions for supporting and rewarding medical staff, with a particular emphasis not only on patient satisfaction but also on employee, as employee relations reflect relationships with the patients [**5**].

It becomes obvious that each employee of a medical organization contributes to a greater or lesser extent, visibly or not, to the positive or negative perception of the quality by patients. The entire staff of the organization is considered a “quality chain” in which each link holds a great importance. If one of the links is weaker, the chain may yield to that point and thus health care service will be compromised [**6**].

Current practice demonstrates that the medical organization’s success crucially depends crucially on the quality of the staff. In most cases, service activities involve the use of a highly skilled workforce, the quality of healthcare professionals leading to quality health services, which in turn affect the overall efficiency of the organization [**7**].

In other words, managers who lead their employees with competence, imagination, and creativity can reach business success in the medical field. In this respect, managers should aim, on principle, at obtaining satisfaction of their employees, to increase productivity and quality of their work by:

• a proper analysis of their own employees’ potential. Expected results should take into account the wishes and needs of the latter;

• motivating employees, based on a quality management by rewarding them according to their performance. An effective system of reward implies an efficient algorithm for the measurement of the employee activity, allowing the identification of those who deserve to be rewarded;

• continuous training of medical personnel. Such education must be constantly updated to be connected with any change in the competitive environment in which the medical organization operates.

**Fig. 1:**
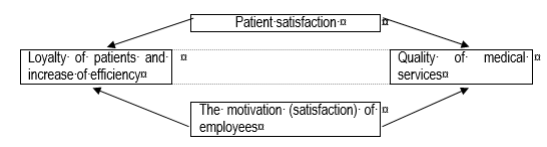
The relationship between patient satisfaction and motivation (satisfaction) of the health care organizations’ employees [**4**]

The healthcare medical staff is responsible for providing quality health services and a high level of patient satisfaction. In this context, the way employees behave and act can increase or diminish the reputation of the sanitary organization (**Fig. 1**).

Finally yet importantly, it must be stressed that an important role in improving the health care services lies with the appropriate selection of the medical staff employed in the organization.

According to the World Health Organization (2000), human resources are the most important component of the health system. Among the new millennium issues relating to human resources are the following:

• low and possibly decreased number of human resources, even in developed countries, so as not to produce enough doctors and other health professionals to fill the existing needs;

• important geographical disparities, particularly between the urban and the rural;

• disparities at the level of training and lack of training or poor training due to the lack of updating the medical practice through training and continuous education;

• pronounced absenteeism caused by inadequate remuneration for the level of responsibility and poor supervision of labor, and cultural barriers in carrying out good work [**8**].

Clearly, the performance of health care organizations is due to the staff employed in both the “front line” - who effectively provide medical service, and the “behind the scenes”, who provide the actual conditions of deployment of health care, while helping create the medical organization’s image among patients [**9**].

## Conclusions

The quality of health services, especially the simultaneous execution and consumption, multitude and perishable nature of the medical staff services are an essential element in the process of providing health services (Author’s note: there are four fundamental characteristics of a service, namely perishable nature, inseparability, intangibility and variability). The promptness and smooth running of the service and the consumer’s satisfaction, with direct results in their health and, respectively in the organization’s ability to attract and keep the clientele depend on the interaction between healthcare professionals and patients.

Besides, there is the premise to exceed them depending on how the medical organization meets the expectations of patients. In its turn, the degree of customer satisfaction is directly proportional to an ongoing effort of the organization to improve health services: each contact with a patient is an opportunity for the latter to feel better than he/she anticipated. Therefore, adequate training, encouraging and motivating the medical staff lead to excellence in medical services, and thus to achieving the expectations of the patients.
